# The impact of age differences in couples on depressive symptoms: evidence from the Korean longitudinal study of aging (2006–2012)

**DOI:** 10.1186/s12888-015-0388-y

**Published:** 2015-02-05

**Authors:** Jae-Hyun Kim, Eun-Cheol Park, Sang Gyu Lee

**Affiliations:** Department of Public Health, Graduate School, Yonsei University, Seoul, Republic of Korea; Institute of Health Services Research, Yonsei University, Seoul, Republic of Korea; Department of Preventive Medicine, Yonsei University College of Medicine, Seoul, Republic of Korea; Department of Hospital management, Graduate School of Public Health, Yonsei University, Seoul, Republic of Korea

**Keywords:** Age differences, Couples, Depressive symptoms

## Abstract

**Background:**

Depression represents one of the most common psychiatric disorders among older adults. Married couples are affected frequently, and psychiatric problems usually affect marital satisfaction. Despite the frequency of such relationships, it appears that very few studies have examined the issues that arise in couples of this type of marriage. Therefore, we investigate whether age differences between couples affect extent of depressive symptoms among older adults.

**Methods:**

Our analysis included 2,881 couples (i.e., 2,881 households) at least 45 years of age at baseline (2006), in addition to 3,033 couples in 2008, 2,772 couples in 2010, and 2,711 couples in 2012. A generalized linear mixed model was used for the data analysis.

**Results:**

When the age difference between husbands and wives was 3 years or less, the estimated severity of depressive symptoms was 0.309 higher (*SE* = 0.084, *p* = 0.000) than that of same-aged couples. When the age gap was 3 years or more, the estimated severity of depressive symptoms was 0.645 higher (*SE* = 0.109, *p* < .0001) than that of same-aged couples. For every 1–2 years extra in age difference between wives and husbands, the estimated severity of depressive symptoms increased by 0.194 (*SE* = 0.082, *p =* 0.018), compared with same-aged couples.

**Conclusions:**

Age differences between husbands and wives impact their relationship, including any particular marital issues encountered.

## Background

Depression represents one of the most common psychiatric disorders, with lifetime prevalence rates of 15% in males and 25% in female and is particularly prevalent among older adults [1].

As an important indicator of mental health, depression is associated closely with lower life satisfaction [2], accompanied often by other mental disorders, physical pains and ailments [3], and burdensome economically [4]. Married couples are affected frequently, and psychiatric problems usually affect marital satisfaction [5].

Numerous empirical studies have provided evidence for the protective effects of marriage on health: married individuals are more likely to be healthier than are widowed, divorced, separated, or never-married individuals [6,7]. Furthermore, married individuals live longer than unmarried individuals [8]. In addition, the impact of the age gap between couples-related studies have been conducted [9]. In United Kingdom, one study, representing one of the first attempts to quantify the influence of spousal age gaps on mortality and longevity, concluded that “conformity to the social norm, of the man being older than his wives, is associated with relatively lower mortality for both parties”. In addition, in Iran, one study [10] about relationship did not found between age difference of couples and marital satisfaction and the maximum age difference between couples was 13 which seem to be culturally acceptable in Iran. Thus, age difference cannot be considered as an effective factor in the patients’ marital satisfaction, family processes and social support in this population.

Differences from this norm, especially when extreme, were associated with higher mortality rates [11]. The researchers speculated that this might be driven by the particular personal characteristics of those who tend to form these unusual partnerships.

When investigating age-heterogamous relationships, researchers have paid particular attention to a number of predictors. Atkinson and Glass [12] attributed changes in this context to an increase in gender equality in Korea. As women become more equal in society, they are less likely to conform to traditional gender roles, therefore foregoing the marital norm in which the male is the older partner.

The importance of factors such as education have also been noted [13,14]. Education appears to play a key role in determining the likelihood that a woman will participate in an age-heterogamous marriage. Social scientists have theorized that an increase in education may be associated with an increased tendency of women to enter heterogeneous relationships [14]. This may be because highly educated women tend to marry later, thus lessening their pool of potential mates and increasing their likelihood of marrying someone younger, as well as to the possibility that they hold more liberal views on marriage.

Several studies have found that poor relationship quality has a greater impact upon wives than husbands [15]. Another important issue concerns cross-spouse effects: the majority of research has focused on the influence of an individual’s marital satisfaction levels on his or her own depressive symptoms. However, the marital satisfaction levels and depression status of wives and husbands are usually correlated [16]. Therefore, it is important to test for cross-spouse effects of marital satisfaction on depression.

Despite the frequency of such relationships, it appears that very few studies have examined the issues that arise in couples of this type of marriage. Although several studies have explored the relationship between marital satisfaction and depression [17], very few that had adjusted for age differences involved older couples, and little is known about partner effects among older populations. Accordingly, factors associated with depression that affect marital satisfaction among older couples remain understudied.

Therefore, we purposed to investigate the relationship between age differences and depressive symptoms in couples in which both partners were at least 45 years of age.

## Methods

### Study sample and design

Data were obtained from the Korean Longitudinal Study of Aging (KLoSA), a nationwide survey of community-dwelling people at least 45 years of age (in 2006), analyzed using multistage stratified cluster sampling. Those results collected between the first and fourth wave of data collection were used. The KLoSA is conducted by the Korea Labor Institute, which aims to devise and implement effective social and economic policies addressing emerging trends in population aging. Additional waves of data collection are undertaken every even-numbered year. The original KLoSA study population comprised South Korean adults, at least 45 years of age, living in 15 large administrative areas.

In the first baseline survey, conducted in 2006, 10,254 individuals among 6,171 households (1.7 per household) were interviewed using the Computer-Assisted Personal Interviewing method. A follow-up survey in 2008 comprised 8,688 subjects, who represented 86.6% of the original panel. The third survey in 2010 used 7,920 subjects, who represented 80.3% of the original panel. Finally, the fourth survey in 2012 involved 7,486 subjects, who represented 76.2% of the original panel.

We restricted our population to married couples experiencing no change in marital status during the previous 8 years and of these participants, we excluded 4,209 subjects that one of them (couples) did not respond to survey in 2006.

We additionally excluded 75 subjects who did not provide the required information.

Our final analysis included 2,881 couples (i.e., 2,881 households) in whom both partners were at least 45 years of age during the 2006 baseline survey; 3,033 couples in 2008; 2,772 couples in 2010; and 2,711 couples in 2012. The KLoSA represents a national public database (http://www.kli.re.kr/klosa/en/about/introduce.jsp), and open data that not included human material or human data. Therefore we don’t need to approve Institutional Review Board.

### Control variables

Four age group categories were used as follows: ≤ 55, 56–65 66–75, and ≥ 75 years. Two employment status categories, “yes” or “no”, were employed; the number of occasions on which couples socialized with friends during the past week was also recorded, according to the following three categories: everyday, sometimes, and never. Economic activity status was divided into two categories: employed and unemployed. Self-rated health was assessed with the question: “How do you usually perceive your health?” The response “very bad” or “bad” indicated “Bad”, and the response “normal”, “good”, or “very good” indicated “Good”, thus dichotomizing the response. Marital satisfaction was assessed with the question: “Are you satisfied with the relationship with your spouse”? The response was ranked from lowest to highest and grouped into three groups (High, Middle, and Low) using the SAS Rank function and the depression status of the partner (spouse effect) were included as covariates in the analyses.

### Depressive symptoms – CESD 10

Several depressive symptoms measurement tools exist, such as the Beck Depression Inventory and the Zung Self-Rating Depression Scale. The 20-item Center for Epidemiological Studies Depression Scale (CES-D), first used in the late 1970s [18], is renowned for its reliability and validity in the context of the general population and primary care patients [19].

The 10-item version of the CES-D (CESD-10), based on the work of Andresen et al., was extrapolated from the original 20-item version by applying item–total correlations and eliminating redundant items [20]. CESD-10 score, the 10-item screening tool we used for depressive symptoms consist of 10 points for the 10-item version. The CES-D has proven to be a useful indicator of depression in older adults.

The CESD-10, which has demonstrated good predictive accuracy in comparison to the full-length 20-item version, assesses three factors: depressive effects (“the blues”, “depressed mood”, “fear”, and “loneliness”), somatic retardation (“bothered”, “sleepy”, “go-getting”, and “attentive”) and positive effects (“happy” and “hopeful”). Depressive symptoms were assessed for 7 days prior to the interview. In this study, we treated depressive symptoms as a continuous measure.

### Analytical approach and statistics

Analysis of variance (ANOVA) and a generalized linear mixed model were used to investigate the impact of age differences between husbands and wives on depressive symptoms. For all analyses, *p* ≤ 0.05 was taken to indicate statistical significance for two-tailed tests. All analyses were conducted using the SAS statistical software package (ver 9.2; SAS Institute Inc., Cary, NC, USA).

### Mixed effects model (SAS® Proc Mixed)

Mixed model was required in order to handle the unbalanced data with correlated outcomes and missing data. In all mixed models presented, only the intercept was allowed to vary between subjects, and the regression slopes were assumed to be fixed effects; random intercept models were applied to our data.

A repeated-measurement model using Proc mixed procedure was performed for this analysis. Depressive symptoms as a continuous variable was the outcome in all mixed models. Covariates of interest from all subjects were added to the model to determine their effects on the increased depressive symptoms. To determine whether the increased depressive symptoms changed over time, we included time (year) in the model as a categorical covariate; the regression coefficient was used to estimate both the change in increased depressive symptoms and independent variables, annually.

## Results

Tables 1 and 2 list the general characteristics of the husbands and wives, respectively (both at baseline [2006]; all characteristics were included as covariates).

**Table 1:** **General characteristics of age difference between husband and wife at baseline (2006)**

	**Total**			**Husband**			
	**N**	**%**	%*	**Mean**	Mean*	SD*	**P-value**
**Huseband-Wife**							<.0001***
**≤ −3**	62	2.15	2.17	0.79	0.99	1.29	
**−2 ~ −1**	145	5.03	5.10	0.25	0.27	0.57	
**0**	251	8.71	8.88	0.22	0.22	0.60	
**+1 ~ +2**	623	21.62	21.05	0.20	0.18	0.63	
**+3 ~ +4**	748	25.96	26.16	0.25	0.24	0.67	
**+5 ~ +6**	533	18.50	18.52	0.20	0.19	0.60	
**≥ +7**	519	18.01	18.12	0.32	0.32	0.77	
**Age**							0.057
**≤55**	898	31.17	33.84	0.16	0.19	0.53	
**56-65**	957	33.22	36.18	0.22	0.23	0.63	
**66-75**	785	27.25	23.86	0.32	0.29	0.76	
**≥75**	241	8.37	6.12	0.50	0.51	0.98	
**Education**							0.188
**≤ Middle school**	1,522	52.83	50.99	0.34	0.33	0.83	
**High school**	886	30.75	32.22	0.17	0.18	0.45	
**≥ College**	473	16.42	16.78	0.12	0.14	0.44	
**Income**							0.104
**Yes**	836	29.02	31.20	0.19	0.22	0.64	
**No**	2,045	70.98	68.80	0.28	0.26	0.70	
**Number of familiarity**							0.029*
**Every day**	958	33.25	31.66	0.22	0.21	0.60	
**Sometimes**	1,675	58.14	59.27	0.24	0.23	0.71	
**Never**	248	8.61	9.07	0.48	0.46	0.77	
**Smoking status**							0.024*
**Never**	1,127	39.12	38.88	0.23	0.21	0.67	
**Former smoker**	703	24.40	23.56	0.31	0.34	0.79	
**Smoker**	1,051	36.48	37.56	0.24	0.24	0.62	
**Alcohol use**							0.103
**No**	696	24.16	23.01	0.24	0.22	0.69	
**Former user**	386	13.40	12.63	0.42	0.42	0.98	
**Yes**	1,799	62.44	64.36	0.22	0.23	0.60	
**Economic activity**							0.127
**Yes**	1,703	59.11	62.47	0.18	0.19	0.60	
**No**	1,178	40.89	37.53	0.36	0.34	0.78	
**Self-rated health**							<.0001***
**Good**	1,470	51.02	53.24	0.11	0.12	0.45	
**Normal**	880	30.54	29.19	0.29	0.30	0.68	
**Bad**	531	18.43	17.58	0.57	0.54	1.02	
**Depression of partner**							<.0001***
**Yes**	792	27.49	26.42	0.64	0.67	0.99	
**No**	2,089	72.51	73.58	0.10	0.10	0.44	
**Marital satisfaction**							0.630
**Low**	83	2.88	2.79	0.49	0.52	0.57	
**Middle**	1,341	46.55	46.96	0.29	0.28	0.76	
**High**	1,457	50.57	50.25	0.20	0.21	0.60	
**Total**	2,881	100.00	100.00	0.25	0.25	0.68	

*p < .05; **p < .01, ***p < .001.

**Table 2:** **General characteristics of age difference between wife and husband at baseline (2006)**

	**Total**			**Wife**			
	**N**	**%**	%*	**Mean**	Mean*	SD*	**P-value**
**Wife-Husband**							<.0001***
**≥ +3**	62	2.15	2.17	1.32	1.70	1.03	
**+1 ~ +2**	145	5.03	5.10	0.46	0.50	0.94	
**0**	251	8.71	8.88	0.34	0.32	0.92	
**−2 ~ −1**	623	21.62	21.05	0.32	0.31	0.76	
**−4 ~ −3**	748	25.96	26.16	0.30	0.27	0.82	
**−6 ~ −5**	533	18.50	18.52	0.38	0.35	0.75	
**≤ −7**	519	18.01	18.12	0.47	0.47	2.33	
**Age**							<.0001***
**≤55**	1,277	44.32	48.23	0.22	0.23	0.64	
**56-65**	930	32.28	34.56	0.34	0.36	0.71	
**66-75**	575	19.96	14.65	0.69	0.74	1.30	
**≥75**	99	3.44	2.55	1.14	1.28	2.15	
**Education**							0.438
**≤ Middle school**	2,026	70.32	68.00	0.47	0.46	1.06	
**High school**	694	24.09	25.90	0.19	0.19	0.56	
**≥ College**	161	5.59	6.10	0.15	0.19	0.44	
**Income**							0.432
**Yes**	407	14.13	15.17	0.28	0.31	0.70	
**No**	2,474	85.87	84.83	0.40	0.39	0.98	
**Number of familiarity**							0.156
**Every day**	1,027	35.65	34.77	0.34	0.32	0.86	
**Sometimes**	1,624	56.37	56.73	0.39	0.39	0.98	
**Never**	230	7.98	8.50	0.56	0.50	1.04	
**Smoking status**							0.029*
**Never**	2,817	97.78	97.84	0.37	0.36	0.91	
**Former smoker**	12	0.42	0.41	1.42	1.13	2.19	
**Smoker**	52	1.80	1.75	0.90	0.99	1.81	
**Alcohol use**							<.0001***
**No**	2,259	78.41	76.76	0.37	0.36	0.92	
**Former user**	66	2.29	2.31	1.14	1.15	1.94	
**Yes**	556	19.30	20.93	0.34	0.34	0.82	
**Economic activity**							0.803
**Yes**	905	31.41	33.31	0.30	0.30	0.79	
**No**	1,976	68.59	66.69	0.42	0.41	1.01	
**Self-rated health**							<.0001***
**Good**	1,169	40.58	42.66	0.14	0.15	0.54	
**Normal**	934	32.42	32.40	0.37	0.39	0.97	
**Bad**	778	27.00	24.94	0.77	0.75	1.22	
**Depression of partner**							<.0001***
**Yes**	576	19.99	19.24	0.95	1.01	1.33	
**No**	2,305	80.01	80.76	0.24	0.23	0.76	
**Marital satisfaction**							0.000**
**Low**	167	5.80	5.59	0.68	0.69	1.13	
**Middle**	1,513	52.52	53.30	0.46	0.46	1.08	
**High**	1,201	41.69	41.11	0.24	0.23	0.68	
**Total**	2,881	100.00	100.00	0.38	0.38	0.94	

*p < .05; **p < .01, ***p < .001.

Data from 2,881 couples at baseline were included. The weighted extent of depressive symptoms for couples with age differences of 3 years or less was 0.99 (*n* = 62 [2.17%], *SD* = 1.29). The weighted extent of depressive symptoms for same-aged couples was 0.22 (*n* = 251 [8.88%], *SD* = 0.60). The weighted extent of depressive symptoms for couples with age differences of 7 years or more was 0.32 (*n* = 519 [18.12%], *SD* = 0.77; Table 1). The weighted extent of depressive symptoms for couples with age differences of 3 years or more was 1.32 (*n* = 62 [2.17%], *SD* = 1.03) in wives and the weighted extent of depressive symptoms for same-aged couples was 0.34 (*n* = 251 [8.88%], *SD* = 0.92). The weighted extent of depressive symptoms for couples with age differences of 7 years or less, was 0.47 (*n* = 519 [18.12%)], *SD* = 2.33; (Table 2). According to our study, 1,277 males felt depressive symptoms (CES-D score ≥ 1) in 2008 compared with subjects with no depressive symptoms (CES-D score = 0) in 2006 and 1,321 females felt depressive symptoms in 2008 compared with subjects with no depressive symptoms in 2006 (Table 3).

**Table 3:** **Incidence in depressive symptoms compared with previous year for 4 years**

		**Following year**					
		**2008**		**2010**		**2012**	
		**N**	**%**	**N**	**%**	**N**	**%**
Previous year (Male)	2006	1,277	49.2				
	2008			500	51.4		
	2010					289	14.0
Previous year (Female)	2006	1,321	66.5				
	2008			473	48.6		
	2010					194	9.8

Table 4 delineates the association between couples’ age differences and the severity of depressive symptoms. For couples with age differences of 3 years or less, the estimated severity of depressive symptoms was 0.309 higher (*SE* = 0.084, *p* = 0.000) than that of same-aged couples. For couples with age differences of 3 years or more, the estimated severity of depressive symptoms was 0.645 higher (*SE* = 0.109, *p* < 0.0001) than that of same-aged couples. For couples with age differences between 1 and 2 years, the estimated severity of depressive symptoms was 0.194 higher (*SE* = 0.082, *p* = 0.018) than that of same-aged couples.

**Table 4:** **Adjusted effect of study variables on depressive symptoms**

	**Husbands**			**Wives**		
	**Estimate**	***SE***	***P*** **-value**	**Estimate**	***SE***	***P*** **-value**
**Husbands-Wives (Wives-Husbands)**						
**≤ −3 (≥ +3)**	0.309	0.084	0.000	0.645	0.109	<.0001
**−2 ~ −1 (+1 ~ +2)**	0.004	0.063	0.955	0.194	0.082	0.018
**0**	ref			ref		
**+1 ~ +2 (−2 ~ −1)**	−0.045	0.045	0.321	0.015	0.059	0.796
**+3 ~ +4 (−4 ~ −3)**	0.024	0.044	0.586	−0.024	0.057	0.668
**+5 ~ +6 (−6 ~ −5)**	−0.054	0.047	0.251	0.075	0.060	0.212
**≥ +7 (≤ −7)**	0.000	0.048	0.996	0.074	0.061	0.222
**Age**						
**≤55**	ref			ref		
**56-65**	−0.038	0.028	0.184	−0.026	0.035	0.464
**66-75**	−0.087	0.035	0.014	0.220	0.048	<.0001
**≥75**	0.108	0.054	0.045	0.524	0.087	<.0001
**Education**						
**≤ Middle school**	0.079	0.030	0.009	0.063	0.062	0.316
**High school**	0.032	0.032	0.318	−0.077	0.065	0.240
**≥ College**	ref			ref		
**Income**						
**Yes**	0.026	0.025	0.296	0.002	0.045	0.967
**No**	ref			ref		
**Frequency of social activities**						
**Everyday**	ref			ref		
**Sometimes**	0.063	0.023	0.005	0.062	0.053	0.247
**Never**	0.160	0.039	<.0001	−0.062	0.056	0.268
**Smoking status**						
**Never**	0.002	0.026	0.947	−0.312	0.113	0.006
**Former smoker**	0.080	0.028	0.005	0.305	0.221	0.168
**Smoker**	ref			ref		
**Alcohol use**						
**No**	ref			ref		
**Former user**	0.167	0.038	<.0001	0.437	0.086	<.0001
**Yes**	−0.002	0.029	0.952	−0.008	0.037	0.824
**Economically active**						
**Yes**	−0.071	0.026	0.006	−0.059	0.035	0.093
**No**	ref			ref		
**Self-rated health**						
**Good**	−0.266	0.031	<.0001	−0.536	0.040	<.0001
**Normal**	−0.205	0.030	<.0001	−0.421	0.037	<.0001
**Bad**	ref			ref		
**Depression in partner**						
**Yes**	0.469	0.024	<.0001	0.811	0.036	<.0001
**No**	ref			ref		
**Marital satisfaction**						
**Low**	0.144	0.059	0.015	0.325	0.063	<.0001
**Medium**	0.069	0.020	0.001	0.189	0.029	<.0001
**High**	ref			ref		
**Year**						
**2006**	ref			ref		
**2008**	1.386	0.038	<.0001	1.688	0.048	<.0001
**2010**	2.586	0.049	<.0001	2.510	0.055	<.0001
**2012**	4.105	0.057	<.0001	3.338	0.061	<.0001

## Discussion

In this study, our primary purpose was to investigate the impact of age differences between couples on depressive symptoms among older adults, using longitudinal models derived from a nationally-representative sample of the general population of South Korea.

The results demonstrated that the age difference between husbands and wives is associated with an increased depressive symptoms in those couples in which the wife is the older partner. Furthermore, the magnitude of depression was higher in the wives compared with the husbands. These associations were independent of variables pertaining to sociodemographics (e.g., age, income status and economic activity status), health risks and behaviors (e.g., number of friends, smoking status, alcohol consumption patterns, and self-rated health), spouse effects, marital satisfaction, and the year in which the data were collected.

In most societies an age difference between the members of a couple is socially normative; typically, the male is older than the female [21]. However, established family dynamics have undergone rapid changes characterized, for example, by a weakening of traditional patriarchy [22]. The extent to which couples in whom the husband is considerably older than his wife are deemed acceptable varies cross-culturally, but relatively large age differences are frequent in patriarchal societies [21]. Greater age differences are often accompanied by concomitant differences in maturity, life experiences, social position and financial resources, which can render relationships inherently unequal and pose a source of risk for women’s health [23-26].

The age difference between married couples has remained relatively stable for several decades in many countries, as noted by Klein [27]. Danish husbands on average are 3 years older than their wives [28]. While the mean age at marriage has increased by approximately 6 years during the twentieth century, especially since the end of the 1960s, the age difference between spouses increased only gradually in the first 50 years, up to the 1950s, but then decreased in the second half of the century [28].

To investigate age differences between couples, three separate theoretical concepts have been posited in recent decades. The most common concept is *homogamy*, or *assortative mating*, which presumes that individuals predisposed through cultural conditioning seek out and marry others like themselves. One assumption here is that a greater age difference is associated with greater marital instability. A further prominent concept is that of the *marriage squeeze*, in which the supply and demand of partners forces individuals to broaden or narrow their age range of acceptable partners. A third, and less common, concept is the *double standard of aging*, which assumes that males are generally less penalized for aging compared with females. This assumption is supported by the greater frequency of partnerships between older males and younger females, and the much greater variability in the ages of males at the time of marriage compared with females [29].

The present results suggest that the wife as the older partner is detrimental for not only her husband, but also for herself. Moreover, controlling for additional covariates (e.g., self-rated health, depression status of the partner, and marital satisfaction) affects the magnitude of depressive symptoms for both husbands and wives; the number of friends did not exhibit an association, however. Nevertheless, one reason cited for gender by age differences in depressive symptoms is social support. A large body of research indicates that women generally have more social contacts than men, but are less likely to be dependent on their social supports [28].

There were a number of strengths and limitations to this study. One strength is that the participants are likely to be representative of the overall population. Furthermore, a large sample size was used, such that the results can be generalized to the general population of older adults in South Korea.

Nevertheless, we do acknowledge a possible sample bias: the respondents’ reports were subjective and potentially affected by false consciousness and recall bias; these data were also not corroborated using medical records, due to the cost and scope of the work that would have entailed. However, our results also suggest that the potential issue of an insufficient length of partnership, to allow for inferences regarding the relationship between depressive symptoms and age gap, was not critical. Finally, although our population was restricted to cases in which there had been no change in marital status for 7 years, the depressive effects of family bereavement (e.g., parents, sons and daughters) may also be a factor in depressive symptoms, but the present study did not directly address the time since widowhood.

## Conclusion

The impact of age difference on the general relationship between husbands and wives and the specific issues that arise for couples were investigated. Several important issues were highlighted by our results. We must remain cognizant of the social stigma that still exists for marriages in which the age differential between the partners is not socially “normative”, and of the greater burden conferred by going against social conventions.
